# Long-Term Outcomes of Prenatally Diagnosed Fetal Hemivertebra: A 15-Year Single-Center Review

**DOI:** 10.3390/children12091236

**Published:** 2025-09-16

**Authors:** Tatiana Costas, María de la O Rodríguez, María Martín Esquilas, Verónica Alarcón, Francisco Javier Goenaga, María Ángeles Cabrero, Ana María Cubo

**Affiliations:** 1 Department of Obstetrics and Gynecology, Hospital Universitario de Salamanca, 37007 Salamanca, Spain; tcostas@saludcastillayleon.es (T.C.); morodriguezm@saludcastillayleon.es (M.d.l.O.R.); mmartinesq@saludcastillayleon.es (M.M.E.); valarcon@saludcastillayleon.es (V.A.); javiergoenaga@saludcastillayleon.es (F.J.G.); macabrero@saludcastillayleon.es (M.Á.C.); 2 Faculty of Medicine, University of Salamanca (USAL), 37007 Salamanca, Spain; 3 Institute of Biomedical Research of Salamanca (IBSAL), 37007 Salamanca, Spain

**Keywords:** hemivertebra, hemivertebrae, prenatal diagnosis, fetal hemivertebra, fetal hemivertebrae

## Abstract

**Highlights:**

What are the main findings?

- Isolated hemivertebra was associated with favorable long-term outcomes, including normal growth and development during childhood.
- Cases with multiple hemivertebrae were frequently accompanied by additional mal-formations or syndromic associations, leading to poorer prognosis and increased need for surgical intervention.

What is the implication of the main finding?

- Early prenatal detection of hemivertebra is crucial to identify associated anomalies and to rule out genetic syndromes, enabling accurate counseling regarding prognosis.
- Differentiating between isolated and multiple hemivertebrae can guide perinatal management and long-term follow-up, as outcomes vary significantly between these groups.

**Abstract:**

Background/Objectives: The primary aim of this study was to describe all cases of fetal hemivertebrae diagnosed prenatally at the Hospital Clínico Universitario de Salamanca over the last 15 years. Additionally, the presence of associated malformations was assessed, pregnancy outcomes were evaluated, and child development results were analyzed in affected cases. Methods: We undertook a prospective observational analysis of all cases (N = 10) of prenatally diagnosed hemivertebrae at our hospital between 2007 and 2022. Postnatal follow-up was performed through telephone interviews and reviewing medical records. Results: Most cases were diagnosed during the second-trimester ultrasound, with the lumbar region being the most frequently affected site (60%). Multiple hemivertebrae were detected in 4 of 10 cases. One case of Marfan syndrome and two cases of VACTERL association (vertebral defects, anal atresia, tracheoesophageal fistula, renal dysplasia, and limb abnormalities) were documented. Six cases presented with additional malformations. Cases involving multiple hemivertebrae (40%) were more likely to be associated with other anomalies and poorer prognoses, while isolated single hemivertebra showed favorable outcomes, with normal development during childhood. Vaginal delivery occurred in six cases, while cesarean sections were performed for standard obstetric indications unrelated to the hemivertebra diagnosis. Conclusions: Prenatal diagnosis of hemivertebra is achievable and holds critical neonatal and postnatal relevance. Hemivertebrae are often linked to additional disorders, including genetic syndromes, and carry significant prognostic implications depending on the associated anomalies and the extent of vertebral involvement.

## 1. Introduction

Hemivertebra is a congenital spinal anomaly that occurs in 1 to 10 per 10,000 live births [1,2] and is characterized by the formation of wedge-shaped vertebrae in the spine [3]. It may affect a single vertebra or multiple vertebrae and is associated with a variable range of other malformations [2,4,5].

The exact etiology of hemivertebra remains unknown. The predominant pathogenic hypothesis suggests that abnormal development of one side of the vertebral body and arch leads to the wedge-shaped deformity. Proposed mechanisms include abnormal distribution of the intersegmental arteries of the spine, nutritional deficiencies such as low zinc intake, or defects in chondrogenesis of vertebral precursors [6]. Other risk factors reported include maternal exposure to high doses of retinoic acid, valproic acid, diabetes, embryonic hypoxia, carbon monoxide, arsenic, ethanol, or hyperthermia [7], as well as rheumatoid arthritis or a smoking habit [8,9].

Hemivertebra can occur as an isolated finding or as part of several syndromic conditions. The most frequently reported associations include VACTERL association (vertebral, anal, cardiac, tracheoesophageal, renal, and limb anomalies), Jarcho–Levin syndrome, Klippel–Feil syndrome, and spondylocostal dysostosis. Hemivertebrae may also be present in chromosomal disorders such as trisomy 18 or 13, and in genetic syndromes affecting skeletal development, including Alagille and DiGeorge syndromes [10]. Therefore, when a hemivertebra is identified prenatally, a thorough evaluation for additional anomalies and syndromic features is essential.

The differential diagnosis of hemivertebra includes other spinal malformations such as partial rachischisis, meningocele, and sacrococcygeal teratomas. Advances in prenatal diagnostic techniques have greatly facilitated the detection of hemivertebra. Ultrasound remains the primary diagnostic tool, although obtaining a clear coronal view of the spine can be challenging due to fetal movements and suboptimal positioning during 2D imaging. Three-dimensional ultrasound may improve assessment by allowing multiplanar evaluation and volume rendering [4,11].

Because hemivertebra is rare, there is limited published information regarding prenatal management, counseling, and postnatal prognosis [1,5,12,13].

This study aimed to describe cases of fetuses diagnosed with hemivertebra at our hospital (Hospital Clínico Universitario de Salamanca) over the past 15 years, and to evaluate associated malformations, gestational outcomes, and postnatal follow-up during infancy.

## 2. Materials and Methods

A prospective and descriptive study was conducted, including 15 years of follow-up, from 2007 to 2022. Only cases with a prenatal diagnosis were included. All patient examinations were carried out by members of the Prenatal Diagnosis Unit at the hospital. Vertebral anomalies were evaluated in real time by a multidisciplinary team comprising obstetricians and pediatric orthopedic surgeons. A complete ultrasound examination of other fetal organs and detailed echocardiography were performed for each fetus. Amniocentesis or CVS for genetic diagnosis was systematically offered to all patients, who decided to accept or decline testing. All patients received appropriate counseling from the same multidisciplinary team. Postnatal outcomes and the progression of these newborns were evaluated by reviewing medical records and conducting regular telephone interviews with their parents.

### 2.1. Inclusion and Exclusion Criteria

All fetuses with a prenatal diagnosis of hemivertebra at our institution between 2007 and 2022 were eligible for inclusion. Cases were included if the diagnosis was confirmed by prenatal imaging and if postnatal follow-up data were available. Exclusion criteria were incomplete records, an absence of confirmatory imaging, loss to follow-up, or refusal of the mother to participate in this study.

### 2.2. Ethical Approval

This study was conducted in accordance with the principles of the Declaration of Helsinki. Ethical approval was obtained from the Salamanca University Hospital Ethics Committee (Protocol code: 2018-01-673, date of approval: 24 January 2018). Written informed consent for participation was obtained from all parents or legal guardians.

### 2.3. Definitions

Hemivertebra was defined as a congenital, wedge-shaped vertebral anomaly resulting from a failure of formation of part of a vertebral body that may occur as a single lesion or involve multiple vertebrae. Associated anomalies were classified according to the organ system involved (cardiac, genitourinary, gastrointestinal, musculoskeletal, central nervous system, and others). The VACTERL association was defined as the non-random co-occurrence of vertebral defects, anal atresia, cardiac malformations, tracheoesophageal fistula with or without esophageal atresia, renal anomalies, and limb abnormalities. Outcomes were categorized as termination of pregnancy, intrauterine fetal death, neonatal death, or survival with or without surgical intervention.

### 2.4. Prenatal Diagnosis

Each case was initially examined with two-dimensional (2D) ultrasound, including sagittal and coronal planes of the fetal spine. In selected cases, three-dimensional (3D) ultrasound was performed to improve characterization of the vertebral anomaly and to assess for associated malformations. All cases were evaluated at the Prenatal Diagnosis Unit of our hospital by specialists (M.O. Rodríguez, A. Cubo, and V. Alarcón), each of whom had more than 15 years of experience in fetal imaging and are recognized as reference experts in this field. Once the diagnosis was established, pediatric orthopedic surgeons were immediately notified and assessed the patient in real time, together with the Prenatal Diagnosis Unit, in order to improve diagnostic accuracy and provide comprehensive counseling to the parents. For genetic diagnosis, amniocentesis or CVS was systematically proposed, and patients were given the choice to either undergo or decline testing. Serial ultrasound examinations were performed throughout pregnancy to monitor each case, evaluate potential changes in prognosis, and detect new associated findings if they appeared. Prenatal diagnosis was confirmed postnatally by neonatal radiographs.

### 2.5. Postnatal Follow-Up

Postnatal follow-up was conducted through a detailed review of medical records complemented by structured telephone interviews with the parents. These interviews were scheduled every six months during the first two years of life and annually thereafter. They focused on the achievement of developmental milestones, psychomotor development, independent ambulation, growth and nutritional status, the need for orthopedic or surgical interventions, the presence of associated malformations, recurrent infections, hospital admissions, school performance, and overall quality of life. This combined approach allowed a comprehensive and long-term assessment of outcomes in affected patients.

## 3. Results

### 3.1. Incidence and Demographics

Among 28,000 newborns during the study period (2007–2022), 10 cases of hemivertebra were identified, resulting in an incidence rate of 0.4 per 1000 live births.

The average maternal age at the time of delivery was 31 years (±7.8 SD). No patient reported exposure to medications, substances, or potentially teratogenic agents during the periconceptional period. There was no reported family history of neural tube or vertebral defects either. All pregnancies occurred spontaneously, without the use of assisted reproductive techniques. The male-to-female ratio was 4:1, with 8 of the 10 fetuses being male and 2 female. A summary of the results is provided in Table 1.

### 3.2. Gestational Age at Diagnosis

**Figure 1:**
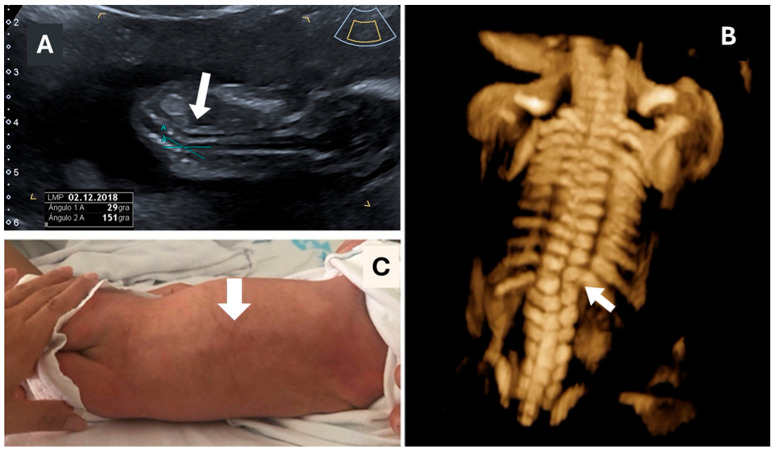
Evolution of a fetus diagnosed with a single hemivertebra (arrows) at the 12-week ultrasound. (**A**) Coronal view of the fetal spine showing distortion of the dorsal spine at 12 weeks. (**B**) Three-dimensional ultrasound at 16 weeks revealing the hemivertebra in the dorsal spine. (**C**) Postnatal images showing the dorsal defect and a change in the spine’s angle. This case was associated with Marfan syndrome, and maternal Marfan syndrome was diagnosed following the fetal diagnosis.

**Figure 2:**
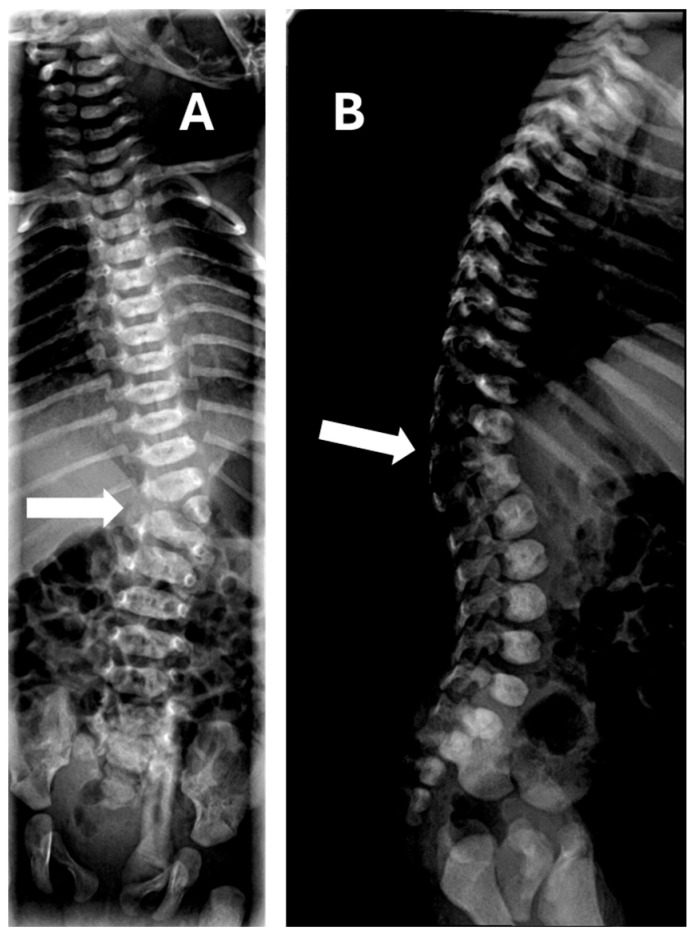
Postnatal radiograph of the spine at 3 months of age: (**A**) anteroposterior view and (**B**) lateral view, showing the presence of a lumbar hemivertebra diagnosed prenatally (arrows).

Most cases (9 of 10) were diagnosed during the second trimester (between 18 and 21 weeks), except for one case, which was diagnosed in the first-trimester ultrasound (Figure 1 and Figure 2).

### 3.3. Genetic Testing and Syndromic Associations

Genetic testing through invasive procedures (amniocentesis or CVS) was offered to all patients, with six cases accepting and undergoing testing. The genetic tests performed included QF-PCR, karyotyping, or array-CGH, all of which showed normal results except for one case that presented with Marfan syndrome.

Regarding associated malformations diagnosed alongside hemivertebra, two cases had a single umbilical artery (SUA), and two pregnancies had renal malformations (one with renal agenesis and the other with an ectopic kidney). One of the cases with a single dorsal hemivertebra was also diagnosed with Marfan syndrome (Figure 1). Both parents were asymptomatic, but further analysis revealed that the mother was also affected. In two cases, diagnosis was associated with VACTERL syndrome (vertebral defects, anal atresia, tracheoesophageal fistula, renal dysplasia, and limb anomalies), one of which included a single umbilical artery and renal agenesis, while the other had an ectopic kidney and partial pulmonary agenesis (both with other associated malformations).

### 3.4. Perinatal Outcomes

The mean gestational age at birth was 38 weeks (SD 2.5), with two preterm births, one at 33 weeks and another at 30 weeks. Both cases involved multiple hemivertebrae and other associated malformations. The average birth weight was 2800 g (SD 556 g). In the group with a single hemivertebra, the average birth weight was 2788 g, while the group with multiple hemivertebrae had an average weight of 2820 g (with only three cases having a birth weight below 2500 g).

### 3.5. Neonatal Outcomes and Follow-Up

The average neonatal follow-up in our cohort was 7.9 years (SD 5.3). Of these ten cases, nine fetuses were live births, while one case resulted in intrauterine demise at 23 weeks. This case occurred in a dichorionic diamniotic twin pregnancy: the fetus with hemivertebra also had a severe ventriculomegaly. The healthy twin had a favorable outcome, being born at 33 weeks of gestation. No patient opted for pregnancy termination.

In our series, most children with isolated single hemivertebra had a good quality of life during childhood, with no need for surgery or signs of disabling conditions. Two-thirds of the cases with multiple hemivertebrae required surgery during childhood or were awaiting surgery due to associated anomalies: case 6 required major surgery due to complications arising from the VACTERL association, which had not been diagnosed prenatally and was identified after birth (imperforate anus), and case 3 required surgery due to talipes equinovarus and spinal instability issues. In the latter case, spinal stability and chronic pain remain ongoing issues requiring medical intervention.

Two of the hemivertebra cases (5 and 9) are currently enrolled in a rehabilitation program and are awaiting surgery due to severe scoliosis or spinal instability. Cases 6 and 9, both involving multiple hemivertebrae and additional associated anomalies, reported a diminished quality of life following surgery.

## 4. Discussion

This study presents a prospective case series analyzing the prenatal aspects and postnatal prognosis of hemivertebrae diagnosed during pregnancy at our hospital over a 15-year period. To our knowledge, this is the first series with a 10-year follow-up in more than 50% of prenatally diagnosed cases, distinguishing it from previously published research, which primarily focused on postnatal diagnoses.

The prevalence of hemivertebrae in our cohort (0.4% of live births; 3.6 per 10,000 live births) is slightly lower than that reported in the literature [1,2], probably because only prenatal diagnosed cases were included in our series. The average maternal age was comparable to other series, such as those by Wax et al. (27.2 years) [13] and Weisz et al. (33 years) [14]. All pregnancies in our series were spontaneous, with one case of dichorionic diamniotic twin pregnancy. This contrasts with the study by Weisz et al., which reported two cases of artificial insemination in twin pregnancies [14]. This finding suggests the need for further exploration into potential relationships between conception methods and the development of hemivertebrae. Similarly, previous studies have suggested that certain maternal conditions, such as pregestational diabetes, rheumatoid arthritis, or a smoking habit, may significantly increase the risk of congenital vertebral anomalies [8,9]. Nonetheless, it should be emphasized that fetal hemivertebra may also arise in the absence of such maternal risk factors, as observed in our series, where none of the patients presented with these conditions. However, given the relatively small sample size of our study, this observation should be interpreted with caution, and it cannot be considered definitive evidence regarding the lack of association with maternal risk factors.

Regarding prenatal findings, the most common ultrasound finding in our series was altered curvature of the fetal spine (Table 1), which aligns with previous reports [2,12,14]. Minor alterations or changes in curvature associated with a single hemivertebra can be difficult to detect. As a result, most series, including ours, report the highest detection rates around 20 weeks [2,3,12,13,14]. Detection before 14 weeks remains a challenge, although advancements in ultrasound, such as 3D ultrasound, are improving early detection [11,15,16,17]. Early detection allows for better perinatal planning and more thorough follow-up.

The most common location site in our series was the lumbar area, followed by the dorsal region (Table 1). This differs from Basude et al. and Wax et al., who reported a higher prevalence in the thoracic region [12,13]. Additionally, we found multiple hemivertebrae in 40% of cases, a percentage similar to other series (25–52%) [12,13,14]. We did not identify an association between the anatomical location and prognosis, which is consistent with the literature.

Hemivertebrae are rarely associated with genetic abnormalities though karyotypic abnormalities; microdeletions and single gene disorders have been described [10,17]. In our series, two cases were associated with VACTERL syndrome, and another case involved Marfan syndrome (Figure 1 and Figure 2), which was also present in the parents, highlighting the importance of genetic screening as, in this case, the finding in the fetus allowed for the study and diagnosis of its parents. Syndromic associations, such as VACTERL, appear to be particularly related to hemivertebrae; our study included one case of thoracic hemivertebra combined with another case of VACTERL syndrome associated with dorsal and lumbar hemivertebrae. While most studies report VACTERL as the most common association [1,2,14], other syndromes have also been described in association with hemivertebrae, including Marfan, Robinow, Conradi–Hünermann, Alagille, Noonan, and Pallister–Hall syndromes, bladder exstrophy sequence, cloacal exstrophy sequence, and Poland syndrome, as well as other chromosomal structural disorders [2,3,10,13,18]. These associations emphasize the importance of genetic screening in prenatal diagnosis of hemivertebrae and the need for systematic prenatal evaluation in suspected cases.

Seventy percent of the cases in our series presented with associated malformations, with renal anomalies being the most common. This slightly differs from other studies that report a higher prevalence of cardiac anomalies [1,2,13]. The high frequency of associated malformations underscores the importance of thorough screening for other structural anomalies in cases of prenatally diagnosed hemivertebrae.

In terms of perinatal outcomes, preterm births were more frequent in cases with multiple hemivertebrae or associated malformations, consistent with the findings of Wax et al. [13], who reported a preterm birth rate of 71.4% in a series of 19 patients. Fetuses with a single hemivertebra had a lower tendency for preterm births. The average birth weight was 2800 g, slightly higher than that reported in other published series [13]. No significant differences were observed between single hemivertebra and multiple hemivertebra cases. The cesarean section rate was similar to that reported in other series (40% versus 50%) [13]. Two cesarean deliveries were performed in cases of multiple hemivertebrae (one of which was a preterm birth), and one in a case of a single hemivertebra. We observed a higher cesarean rate irrespective of the hemivertebra diagnosis, likely due to the increased incidence of preterm birth in this group or a greater tendency for fetal malpresentation or malposition.

Postnatal prognosis was influenced by the number of affected vertebrae and associated malformations. Prenatal deaths associated with this condition were rare in the reviewed series [12,14], as in our cohort. These were typically associated with other more significant malformations or anomalies (such as severe hydrocephalus in our series). In our cohort, 40% of cases had a good prognosis, while the remainder presented with significant complications or required early surgical intervention, reflecting the heterogeneity of the clinical course described in the literature [10,18]. We did not find a specific association with a particular anatomical location [12,14].

Finally, predicting prognosis in this malformation is complex, as the progression of hemivertebra is highly variable: in 25% of cases, there is no scoliosis, 50% exhibit slow development, and 25% require surgery in the early years of life [19]. In our series, 40% of cases had a good prognosis, while those with a worse prognosis had multiple hemivertebrae and other associated disorders.

This study has strengths and limitations. Its main strength is the long-term follow-up of prenatally diagnosed hemivertebra cases, providing valuable insights into postnatal outcomes in a rare condition. The main limitations are the small sample size, largely conditioned by the rarity of this condition, the single-center design, and the absence of a control group, which may restrict the generalizability of the findings.

## 5. Conclusions

Our study highlights the importance of early prenatal diagnosis, genetic screening, and systematic evaluation of associated malformations in cases of hemivertebrae. Early detection during pregnancy is crucial for ensuring adequate postnatal care and timely follow-up. Although syndromic associations are uncommon, they should be carefully evaluated, as should associated malformations, which have a direct impact on prognosis. Cases of isolated hemivertebra generally have a favorable prognosis, with normal growth and development during childhood.

## Figures and Tables

**Table 1:** Description of the characteristics and outcomes of patients included in this study.

Patient Number	Maternal Age (Years)	GA at Diagnosis (Weeks)	Amniocentesis /CVS	Localization	Single vs. Multiple	Fetal Gender	Syndromic Association	Other Malformations	Type of Delivery	Gestational Age at Delivery (Weeks)	Weight at Delivery (g)	Follow Up (Years)	Gestational Outcome	Evolution
1	40	12	Yes	Dorsal	Single	Female	Marfan ^a^	No	CS	40	2695	1	Live born	Asymptomatic, normal child life
2	31	20	Yes	Lumbar	Single	Male	No	No	VD	36	2200	10	Live born	Asymptomatic, normal child life
3	20	20	Yes	Lumbar + sacrum	Multiple	Male	No	Thallus foot	VD	30	3160	11	Live born	Trauma surgery at 7 years
4	36	20	No *	Sacrum	Single	Male	Unknown	No	VD	39	3260	15	Live born	Asymptomatic, normal child life
5	34	20	Yes	Lumbar	Single	Male	No	No	VD	37	2940	13	Live born	In a rehabilitation program, awaiting spinal surgery
6	41	20	Yes	Dorsal + lumbar	Multiple	Female	VACTERL	Renal agenesis, SUA	VD	39	3620	9	Live born	Vesico-ureteral fistula and anal surgeries
7	36	20	No *	Lumbar	Single	Male	Unknown	No	VD	40	3320	0.2	Live born	Asymptomatic, normal child life
8 ^b^	20	20	Yes	Dorsal	Single	Male	No	Hydrocephalus, SUA	-	-	-	-	IUD	-
9	22	18	No *	Thoracic	Multiple	Male	VACTERL	Ectopic kidney, partial pulmonary agenesis	CS	33	2000	4	Live born	In a rehabilitation program, awaiting spinal surgery
10	38	19	No *	Lumbar	Multiple	Male	No	No	CS	38	2500	7	Live born	Asymptomatic, normal child life

* Patient declined test; ^a^ Maternal Marfan syndrome was diagnosed after the syndrome was found in the fetus; ^b^ Bicorial, biamniotic pregnancy. First twin affected, intrauterine demise. Second twin healthy, born at 33 weeks; VACTERL: vertebral defects, anal atresia, tracheoesophageal fistula, renal dysplasia, and limb abnormalities; GA: gestational age; CVS: chorionic villus sampling; VD: vaginal delivery; CS: cesarean section; SUA: single umbilical artery; IUD: intrauterine demise.

## Data Availability

Original contributions presented in this study are included in the article. Further inquiries can be directed to the corresponding author.

## Abbreviations

The following abbreviations are used in this manuscript:

Array-CGH	Array Comparative Genomic Hybridization
CS	Cesarean Section
CVS	Chorionic Villus Sampling
GA	Gestational Age
IUD	Intrauterine Demise
QF-PCR	Quantitative Fluorescent Polymerase Chain Reaction
SUA	Single Umbilical Artery
VACTERL	Vertebral Defects, Anal Atresia, Tracheoesophageal Fistula, Renal Dysplasia, and Limb Abnormalities
VD	Vaginal Delivery

## References

[1] Goldstein I., Makhoul I.R., Weissman A., Drugan A. (2005). Hemivertebra: Prenatal Diagnosis, Incidence and Characteristics. Fetal Diagn. Ther..

[2] Forrester M.B., Merz R.D. (2006). Descriptive Epidemiology of Hemivertebrae, Hawaii, 1986–2002. Congenit. Anom..

[3] Varras M., Akrivis C. (2010). Prenatal diagnosis of fetal hemivertebra at 20 weeks’ gestation with literature review. Int. J. Gen. Med..

[4] Volpe N., Migliavacca C., Dall’Asta A., Kaihura C.T., Ghi T., Frusca T. (2020). Prenatal Diagnosis of Fetal Multiple Hemivertebrae: The Importance of 3D Ultrasound Assessment. J. Matern. Neonatal Med..

[5] Upasani V.V., Ketwaroo P.D., Estroff J.A., Warf B.C., Emans J.B., Glotzbecker M.P. (2016). Prenatal Diagnosis and Assessment of Congenital Spinal Anomalies: Review for Prenatal Counseling. World J. Orthop..

[6] Tanaka T., Uhthoff H.K. (1981). The Pathogenesis of Congenital Vertebral Malformations. A Study Based on Observations Made in 11 Human Embryos and Fetuses. Acta Orthop. Scand..

[7] Alexander P.G., Tuan R.S. (2010). Role of Environmental Factors in Axial Skeletal Dysmorphogenesis. Birth Defects Res. Part C-Embryo Today Rev..

[8] Raitio A., Heiskanen S., Syvänen J., Leinonen M.K., Kemppainen T., Löyttyniemi E., Ahonen M., Gissler M., Helenius I. (2023). Maternal Risk Factors for Congenital Vertebral Anomalies: A Population-Based Study. J. Bone Jt. Surg..

[9] Hesemann J., Lauer E., Ziska S., Noonan K., Nemeth B., Scott-Schwoerer J., McCarty C., Rasmussen K., Goldberg J.M., Sund S. (2013). Analysis of Maternal Risk Factors Associated with Congenital Vertebral Malformations. Spine.

[10] Powel J.E., Sham C.E., Spiliopoulos M., Ferreira C.R., Rosenthal E., Sinkovskaya E.S., Brown S., Jelin A.C., Al-Kouatly H.B. (2022). Genetics of Non-Isolated Hemivertebra: A Systematic Review of Fetal, Neonatal, and Infant Cases. Clin. Genet..

[11] Wen Y., Xiang G., Liang X., Tong X. (2018). The Clinical Value of Prenatal 3D Ultrasonic Diagnosis on Fetus Hemivertebra Deformity- A Preliminary Study. Curr. Med. Imaging Rev..

[12] Basude S., Mcdermott L., Newell S., Wreyford B., Denbow M., Hutchinson J., Abdel-Fattah S. (2015). Fetal Hemivertebra: Associations and Perinatal Outcome. Ultrasound Obstet. Gynecol..

[13] Wax J.R., Watson W.J., Miller R.C., Ingardia C.J., Pinette M.G., Cartin A., Grimes C.K., Blackstone J. (2008). Prenatal Sonographic Diagnosis of Hemivertebrae: Associations and Outcomes. J. Ultrasound Med..

[14] Weisz B., Achiron R., Schindler A., Eisenberg V.H., Lipitz S., Zalel Y. (2004). Prenatal Sonographic Diagnosis of Hemivertebra. J. Ultrasound Med..

[15] Yulia A., Pawar S., Chelemen O., Ushakov F., Pandya P.P. (2020). Fetal Hemivertebra at 11 to 14 Weeks’ Gestation. J. Ultrasound Med..

[16] Chen M., Chan B., Lam T.P.W., Shek T., Lee C.P., Tang M.H.Y. (2007). Sonographic Features of Hemivertebra at 13 Weeks’ Gestation. J. Obstet. Gynaecol. Res..

[17] Kalache K.D., Bamberg C., Proquitté H., Sarioglu N., Lebek H., Esser T. (2006). Three-Dimensional Multi-Slice View: New Prospects for Evaluation of Congenital Anomalies in the Fetus. J. Ultrasound Med..

[18] Lemire G.T., Beauregard-Lacroix É., Campeau P.M., Parent S., Roy-Beaudry M., Soglio D.D., Grignon A., Rypens F., Wavrant S., Laberge A.M. (2020). Retrospective Analysis of Fetal Vertebral Defects: Associated Anomalies, Etiologies, and Outcome. Am. J. Med. Genet. Part A.

[19] Caredda M., Bandinelli D., Falciglia F., Giordano M., Aulisa A.G. (2022). The Conservative Treatment of Congenital Scoliosis with Hemivertebra: Report of Three Cases. Front. Pediatr..

